# Neurological and systemic manifestations of cerebral autosomal dominant arteriopathy with subcortical infarcts and leukoencephalopathy (CADASIL): a nationwide study in Korea

**DOI:** 10.1002/alz70860_098164

**Published:** 2025-12-23

**Authors:** Young Hee Jung

**Affiliations:** ^1^ Sacred Heart Hospital, College of Medicine, Hallym University, Anyang, Gyeonggido, Korea, Republic of (South)

## Abstract

**Background:**

This study investigated the epidemiological and clinical features of Cerebral Autosomal Dominant Arteriopathy with Subcortical Infarcts and Leukoencephalopathy (CADASIL) in South Korea, comparing patients to age‐ and sex‐matched controls.

**Methods:**

We analyzed newly diagnosed CADASIL patients aged over 20 years and matched controls from the National Health Information Database (2002–2022). Cumulative incidence by age, sex, and region was calculated, and the prevalence of neurological and systemic conditions was compared. Neurological conditions included Alzheimer's disease (AD), vascular dementia (VaD), epilepsy, mild cognitive impairment, stroke, migraine, and Parkinson's disease. Systemic conditions included diabetes mellitus (DM), hypertension (HTN), hypercholesterolemia, atrial fibrillation/flutter, myocardial infarction, cancer, and depression.

**Results:**

A total of 816 CADASIL patients (mean age: 56.8 ± 15.2 years; 48.2% male) and 816 controls were included. The cumulative incidence of CADASIL per 100,000 persons was 1.86 (95% CI: 1.85–1.87), peaking at 60–69 years. Incidence was higher in men except for those over 70 years, with Jeju showing the highest regional incidence (39.67 per 100,000; 95% CI: 37.84–41.49).

The prevalence of AD (33.1% vs. 20.0%) and VaD (85% vs. 5%) was significantly higher in the CADASIL group than in controls (*p* <0.001). Epilepsy, migraine, and stroke (hemorrhagic: 9.8% vs. 6.4%, ischemic: 69.7% vs. 24.1%, *p* <0.001) were also more common. Systemic conditions such as DM (79.2% vs. 69.0%), hyperlipidemia (92.9% vs. 78.1%), and depression (60.8% vs. 44.9%) were significantly more frequent in the CADASIL group (all *p* <0.001). Disease onset occurred earlier in CADASIL patients compared to controls.

**Conclusion:**

This study highlights the substantial disease burden of CADASIL in Korea, with higher prevalence of neurological and systemic diseases and earlier disease onset in CADASIL patients, underscoring the need for early diagnosis and comprehensive management strategies.

